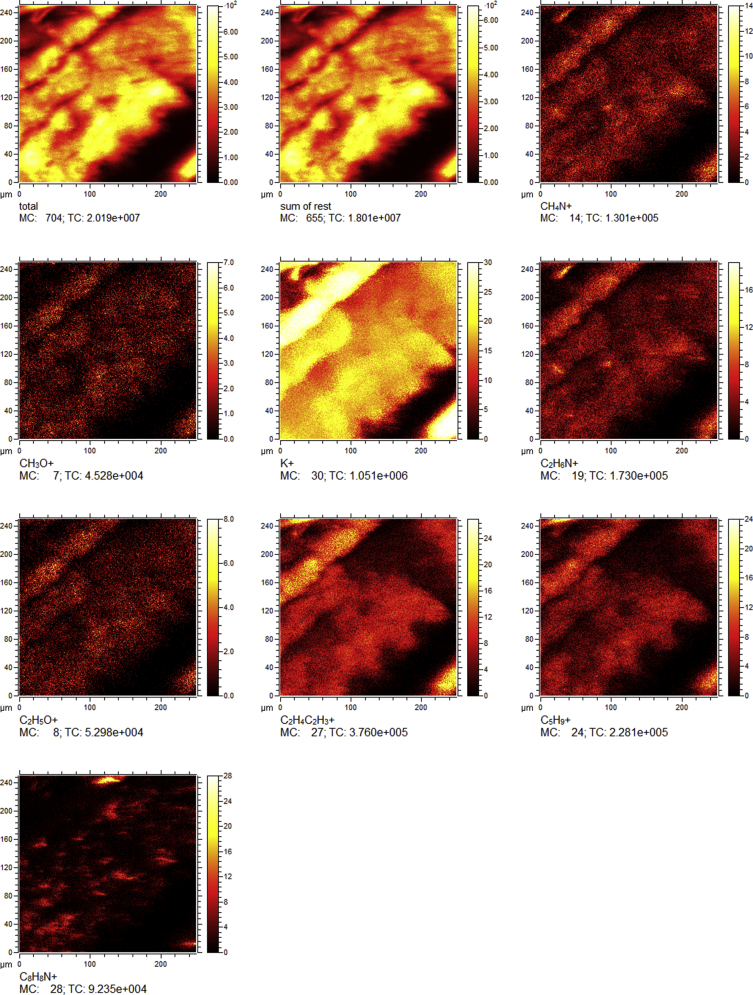# Baseline characterization data for raw rice husk

**DOI:** 10.1016/j.dib.2019.104219

**Published:** 2019-07-16

**Authors:** Praveen Kolar, Han Jin

**Affiliations:** North Carolina State University, Biological and Agricultural Engineering, 278 Weaver Labs, Campus Box 7625, 27695-7625, Raleigh, NC, USA

**Keywords:** Rice husk, Characterization, Acid value, PZC, XPS, SEM-EDX, Specific surface area

## Abstract

There is a significant interest in using agricultural wastes such as rice husk as a precursor for the synthesis of adsorbents and catalysts. In this article, readers will find valuable baseline characterization data related to physical and chemical properties of raw rice husk including BET specific surface area, acid value, the point of zero charge, elemental analysis, Time-of-Flight Secondary Ion Mass Spectrometric Analysis X-Ray Photoelectron Spectroscopic Analysis, and Scanning Electron Microscope-Energy Dispersive Spectroscopic Analysis. It is expected that the baseline raw data presented in this article will be useful for researchers around the world who are working on chemically modifying rice husk for valorizing them for applications in adsorption, catalysis, and energy storage.

**Table:** Specifications Table

Subject area	*Agriculture and Environment & Chemical Engineering*
More specific subject area	*Adsorption and Waste Valorization*
Type of data	*Table, images, plots, and spreadsheet*
How data was acquired	*BET Surface Area Analyzer, Variable Pressure Scanning Electron Microscope-EDX (Hitachi S–3200 N), X-Ray Photoelectron Spectroscopy (a SPECS FlexMod-PHOIBIS 150), Time-of-Flight-Secondary Ion Mass Spectroscopy (TOF-SIMS) (ION TOF, Inc.), and Wet Chemistry.*
Data format	*Raw data, processed and plotted data, and images*
Experimental factors	*The samples were analyzed as received*
Experimental features	*Rice husk samples were analyzed for physical and chemical properties to obtain baseline data.*
Data source location	*North Carolina State University, Raleigh, NC, USA.*
Data accessibility	*All data associated with this article are attached.*
Related research article	*Y. Zhu, P. Kolar, Investigation of adsorption of p-cresol on coconut shell-derived activated carbon. Journal of the Taiwan Institute of Chemical Engineers, 68 (*2016*) 138–146*. https://doi.org/10.1016/j.jtice.2016.07.044.

**Table:** 

**Value of the data** • There is a significant interest in using rice husk as a precursor for the synthesis of adsorbents and catalysts for the removal of pollutants from water and for use in energy storage devices. • Baseline data will provide valuable information on the physical and chemical properties of rice husk. • Other researchers can use the baseline data to suitably modify the rice husk depending on the target application. • The baseline data of raw husk can also be used to compare the physical and chemical properties of the modified rice husk to draw understand the changes in surface properties due to physical and chemical modification.

## 1 Data

**Fig. 1:**
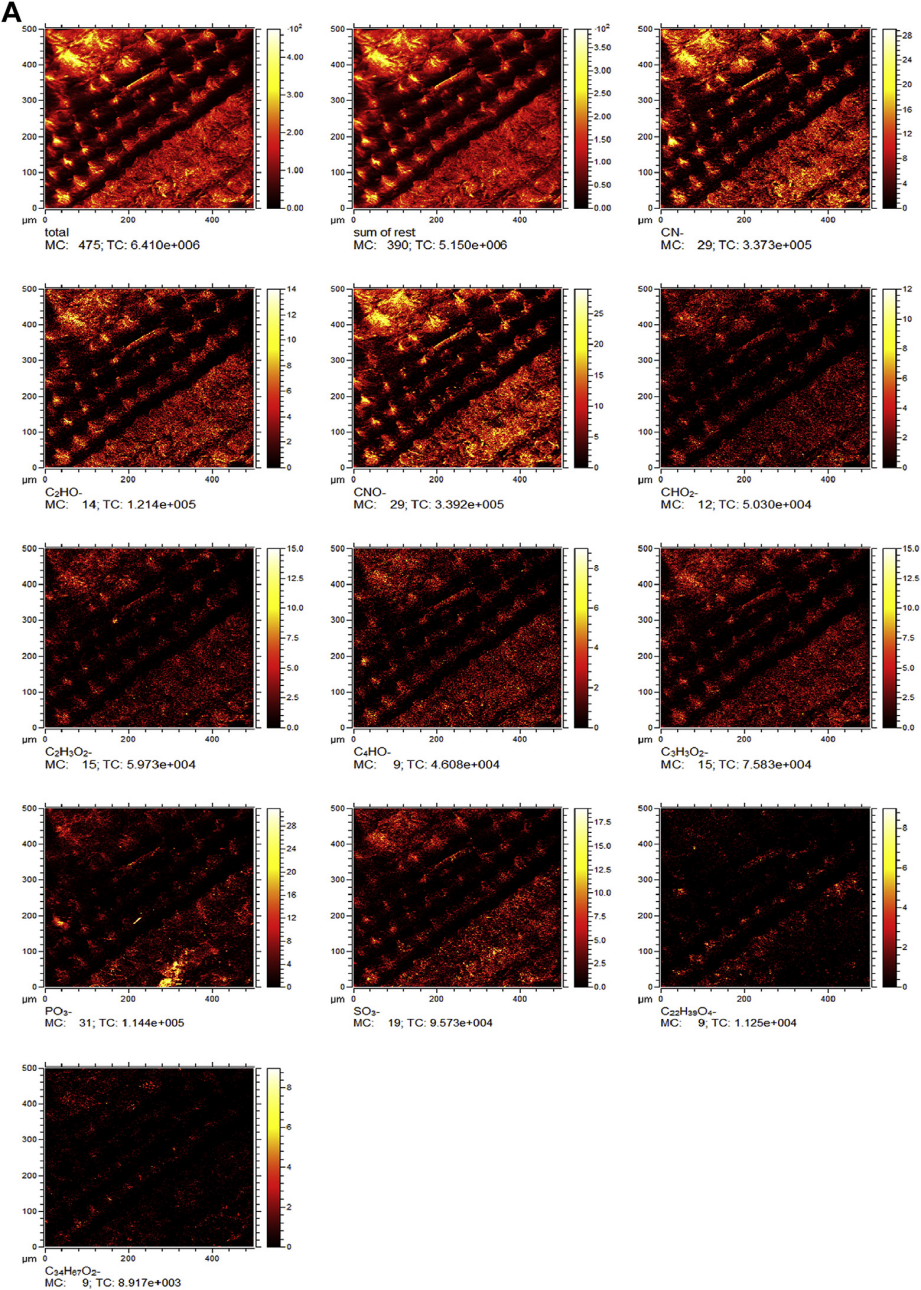

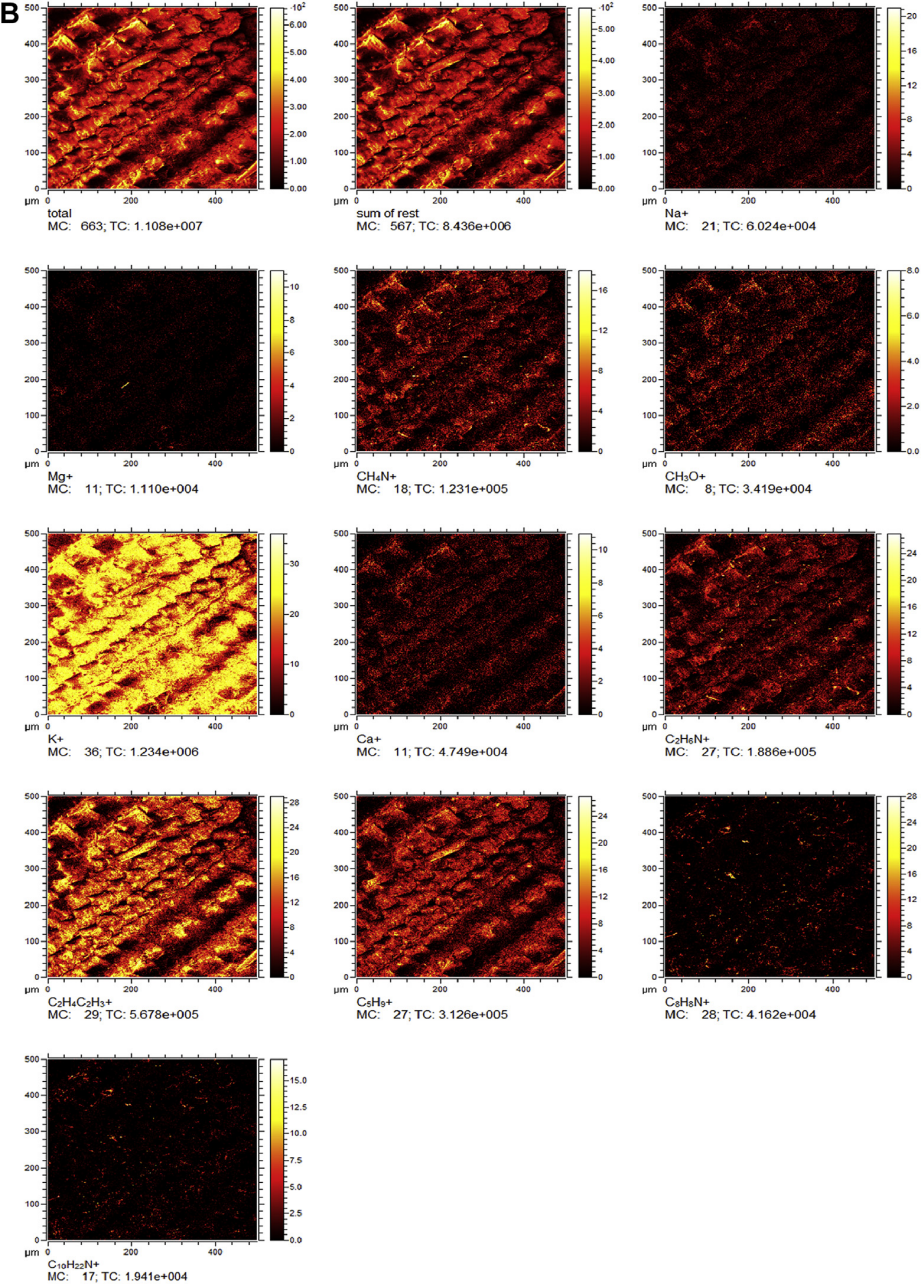
A. High resolution negative ion spectra of rice husk. B. High resolution positive ion spectra of rice husk.

**Fig. 2:**
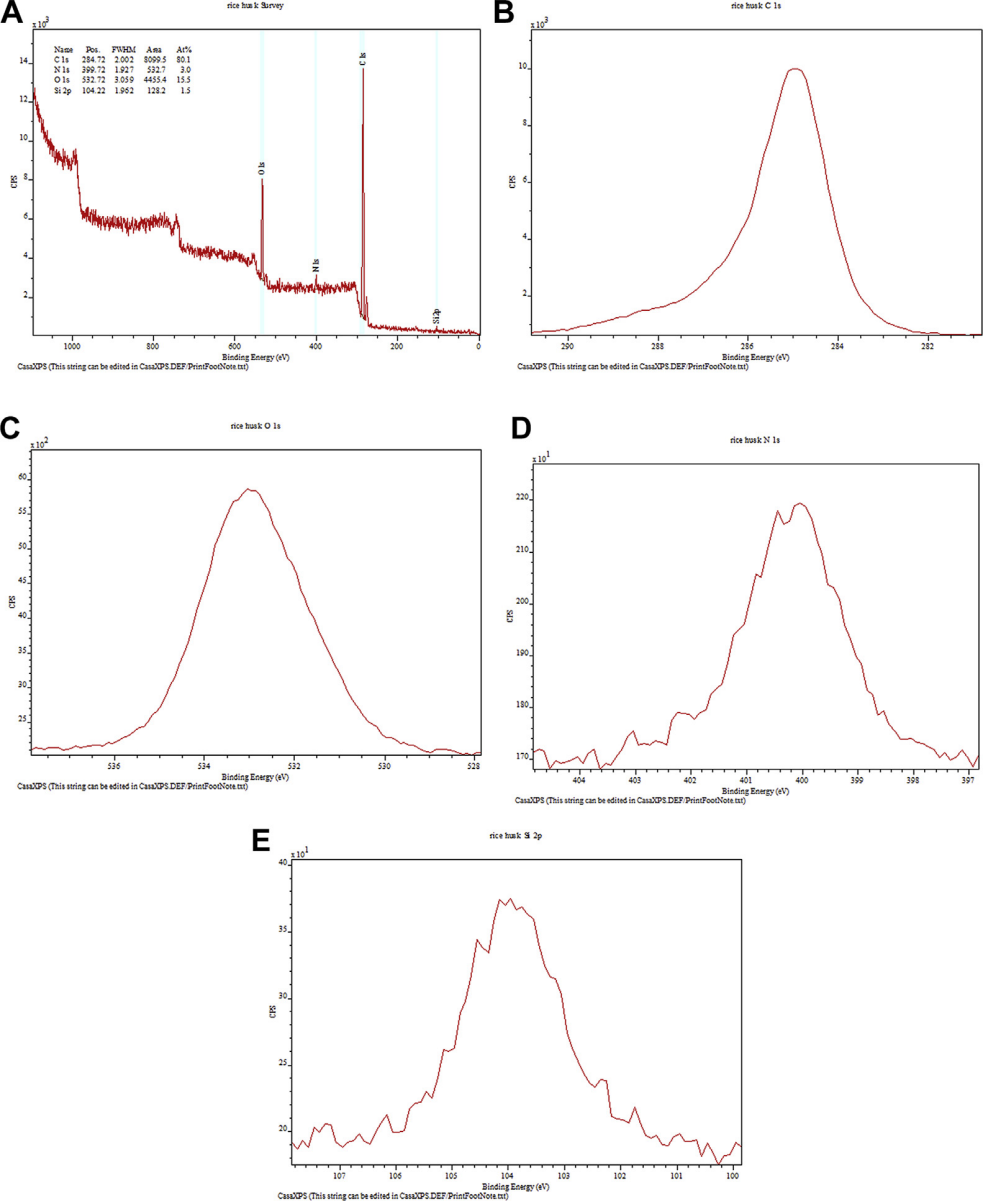
A. XPS Survey spectrum of raw rice husk. B. XPS spectrum of C 1s collected from raw rice husk. C. XPS spectrum of O 1s collected from raw rice husk. D. XPS spectrum of N 1s collected from raw rice husk. E. XPS spectrum of Si 2p collected from raw rice husk.

**Fig. 3:**
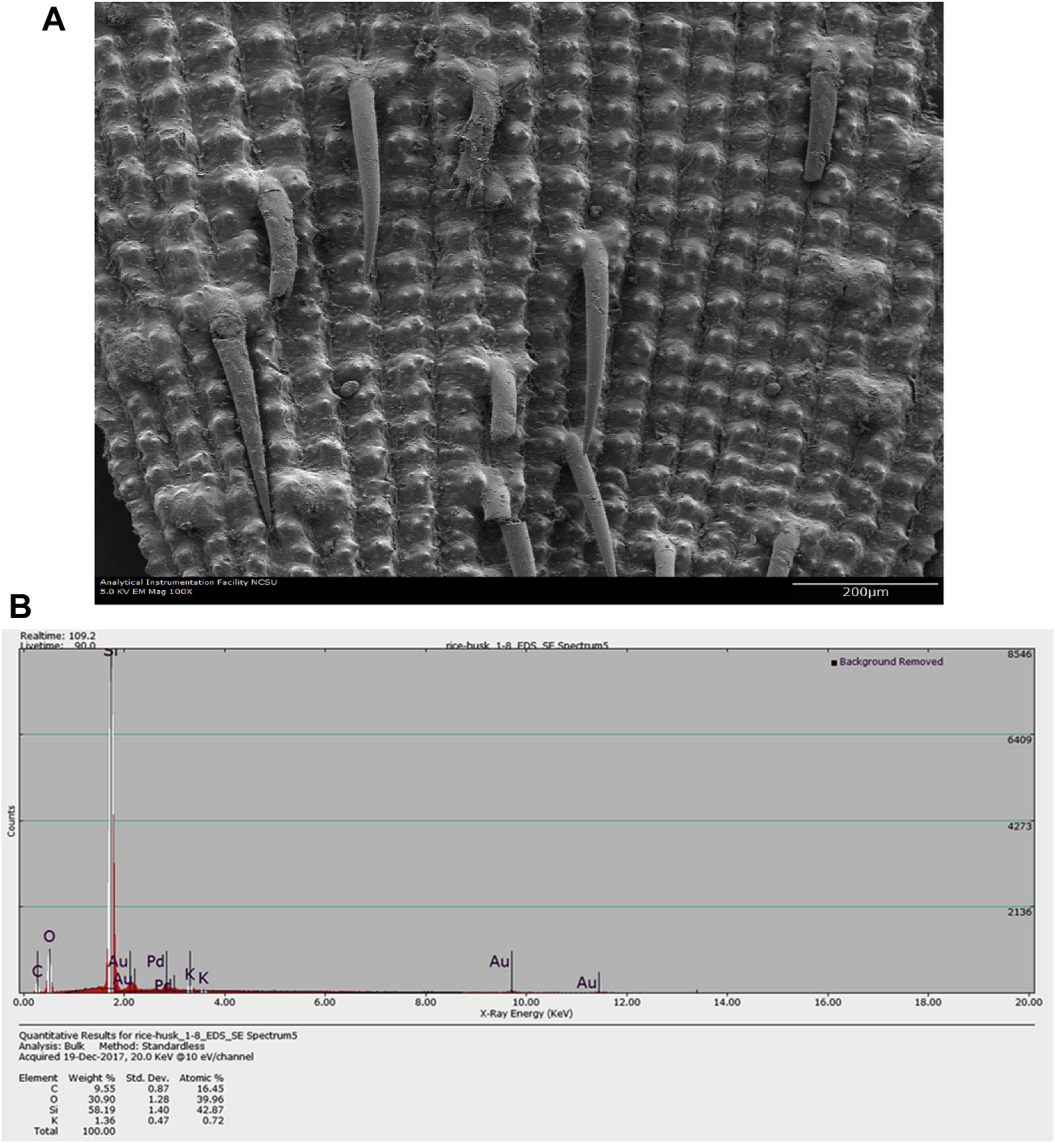

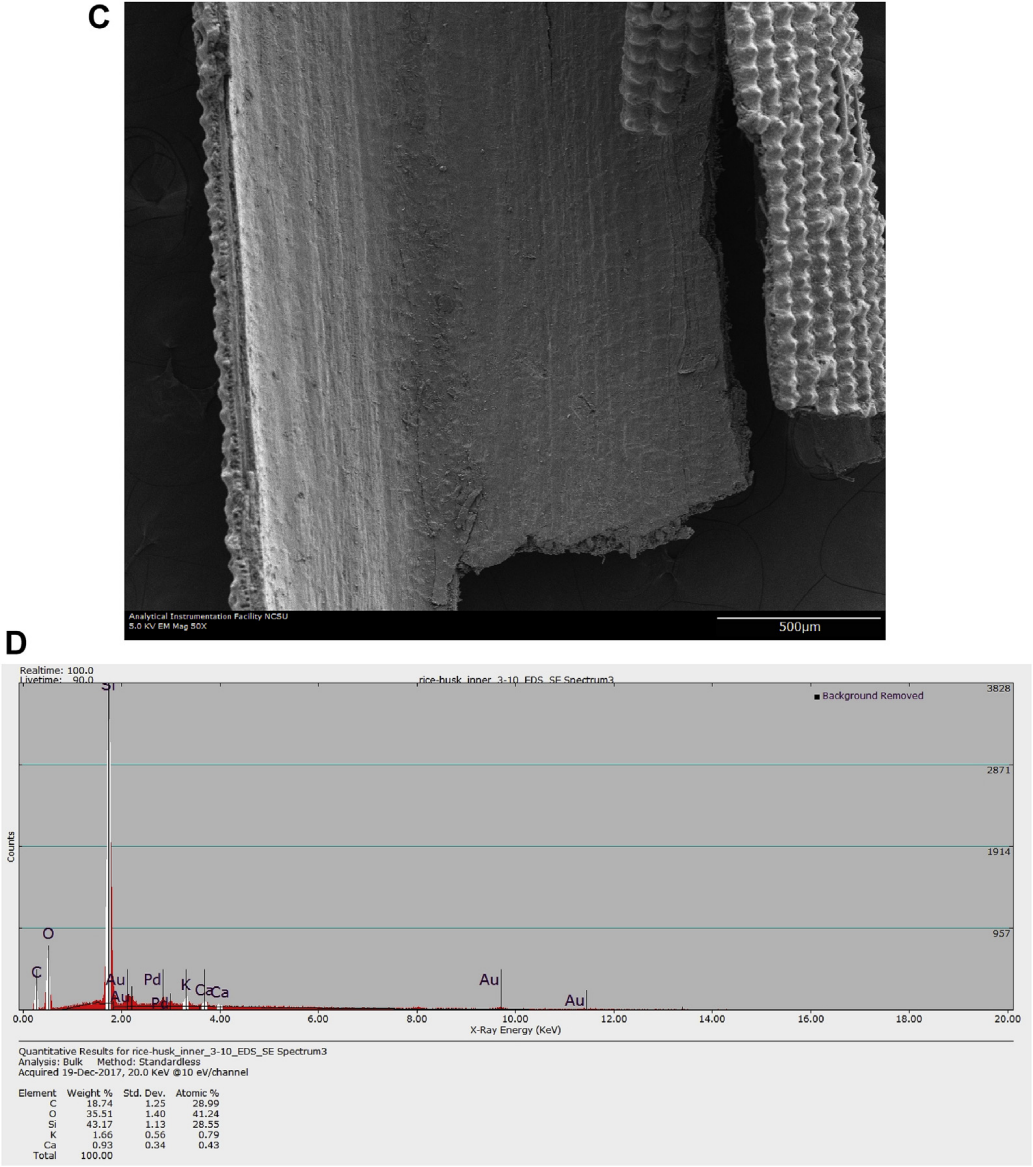
A. Micrograph of outer surface of rice husk. B. EDX Spectrum of outer surface of rice husk. C. Micrograph of inner surface of rice husk. D. EDX Spectrum of inner surface of rice husk.

Raw rice husk was analyzed to collect benchmark data on surface physical and chemical properties. The BET specific surface area, acid value, and point of zero charge are presented in Table 1. The pH (acid value) was found to be 6.91 indicating that the surface is balanced in neutral solutions. The data suggested that the change in solution pH may play a significant role in the surface chemistry of the rice husk. The measured PZC of 6.18 indicated that the raw rice husk is negatively charged in aqueous systems whose pH is greater than 6.18 and positively charged below a pH of 6.18. Considering that rice husk will interact with cations beyond a pH of 6.18 and anions when pH is below 6.18, these data can have significant implications in the selection, design, and analysis of adsorption systems. The high-resolution TOF-SIMS spectra and the fragmented positive and ions are shown in Fig. 1(A and B) The XPS survey spectral data (Fig. 2A) revealed that the surface of the raw rice husk consisted mainly of carbon, oxygen, nitrogen, and silica. The peaks pertaining to C 1s, O 1s, N 1s, and Si 2p are presented separately in Fig. 2(B–E). The surface topographical features including EDX data is presented in fig. 3(A–D), which revealed that the rich husk surface is equipped with a smooth inner surface and a systematically undulating outer surface consisting of carbon, oxygen, silica, and traces of potassium and calcium. In addition, all the raw data including additional SEM micrographs are attached separately. We expect that researchers can access and use these raw data as they see fit.

**Table 1:** Characterization data of raw rice husk.

Property	Measured value
Elemental composition^a^	C, O, N, Si, K, and traces of Ca, Na, S, and Mg
Point of zero charge (PZC)	6.18
Acid value	6.91 ± 0.23
BET surface area (m^2^ g^−1^)	0.1403

a From XPS, EDX, and TOF-SIMS Data.

## 2 Materials and methods

### 2.1 Specific surface area (SSA)

The SSA was determined using a Micrometrics Gemini VII 2390p analyzer using the standard nitrogen adsorption-desorption technique. The rice husk samples were degassed (150 °C) under nitrogen flow for 3 h. Subsequently, the BET analysis was performed.

### 2.2 Acid value

0.4 g of rice husk was contacted with 20 mL of deionized water overnight as described by Ref. [1]. Subsequently, the rice husk was filtered out and the pH of the solution was determined.

### 2.3 Point of zero charge (PZC)

**R**ice husk (0.15 g) was equilibrated for 48 hours in 50 mL of 0.01 M sodium chloride solutions adjusted to pH values of 2–10 using either 0.1 M HCl or 0.1 M NaOH as described by Ref. [2]. After filtering the rice husk samples out the final pH was measured. The PZC of rice husk was determined by identifying the point of intersection of pH_initial_ = pH_final_ line with the pH_initial_ vs pH_final_ curve.

### 2.4 X-ray photoelectron spectroscopy (XPS)

Samples of rice husk were mounted on a SPECS FlexMod XPS unit equipped with a hemispherical analyzer PHOIBIS 150 and Mg K alpha (1254 eV) X-ray source. The sample chamber was maintained in the order of 10^−10^ mbar during the data collection. Adventitious Carbon (C1s @ 285.0 eV) was used as a reference for energy calibration.

### 2.5 Time of flight-secondary ion mass spectroscopy (TOF-SIMS)

The data was collected using a TOF SIMS V (ION TOF, Inc. Chestnut Ridge, NY) instrument equipped with a Bi_n_^m+^ (n = 1–5, m = 1, 2) liquid metal ion gun, Cs^+^ sputtering gun which were angled at 45° to the sample surface normal while maintaining the pressure inside the chamber below 5.0 × 10^−9^ mbar. The high-resolution data was collected via the pulsed Bi^3+^ primary ion beam (25 kV). For calibrating the negative secondary ion spectra, C^−^, O^−^, OH^−^, and C_n_^−^, were employed. Similarly, for the positive ion spectra calibration H^+^, C^+^, C_2_H_3_^+^, C_3_H_5_^+^, and C_4_H_7_^+^ [3]

### 2.6 Scanning electron microscopy (SEM)

Rice husk samples were placed on a sticky tab and sputter coated with 60/40 Au/Pd for 5 min @ 7 nm min^−1^. Subsequently, the surface morphology (texture) and chemical composition (via EDX) were analyzed using a Hitachi S3200 N variable pressure SEM via a 5 keV and 20 keV electron beam for imaging and EDX, respectively, as described in Ref. [4].

## Figures and Tables

**Figure:**
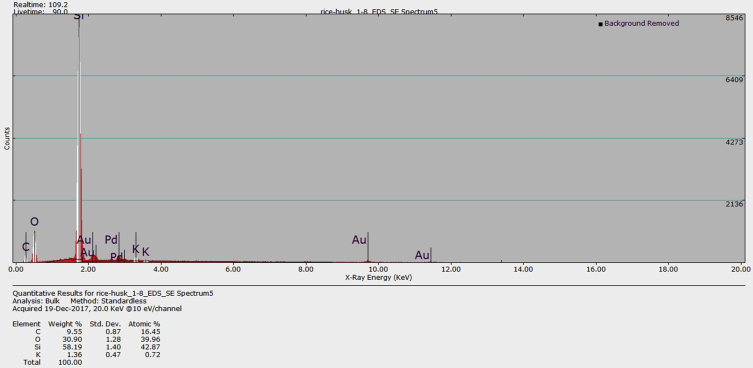


**Figure:**
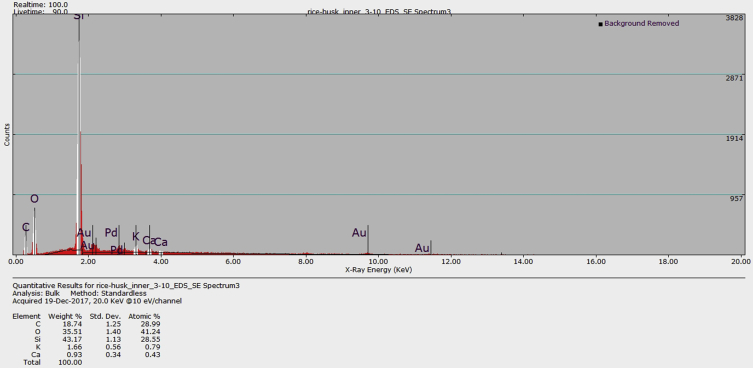


**Figure:**
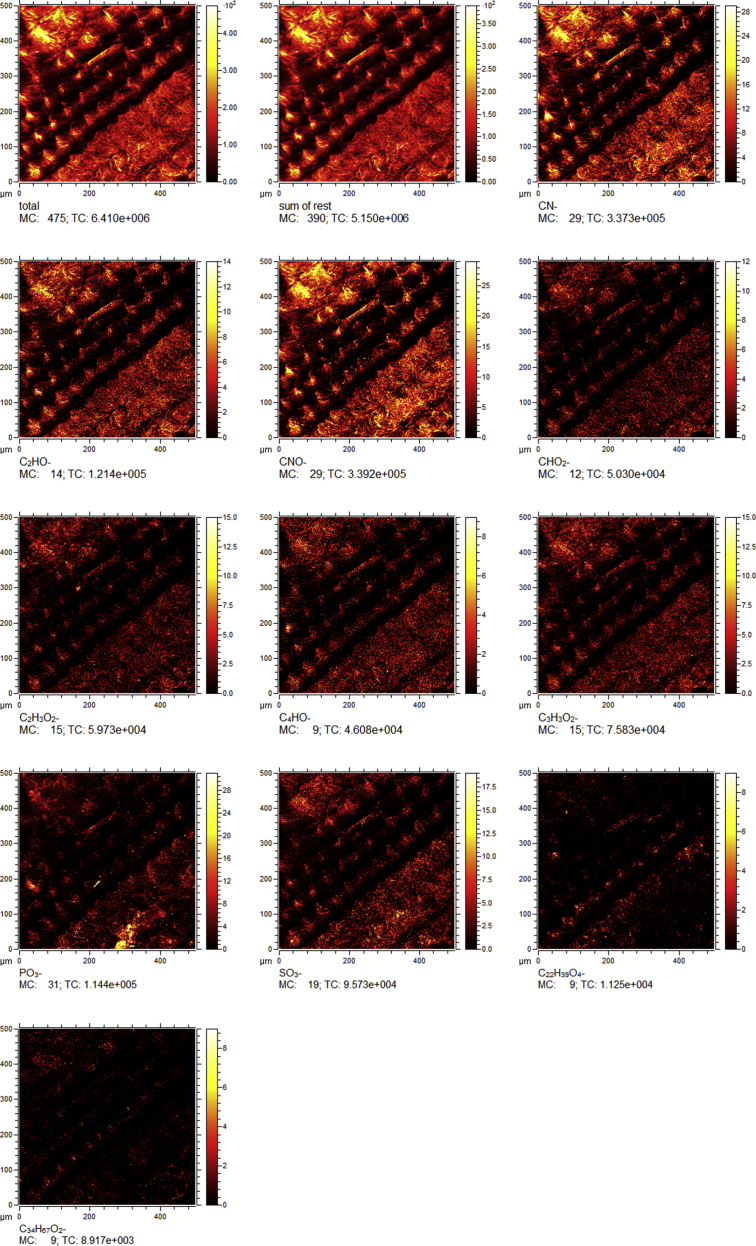


**Figure:**
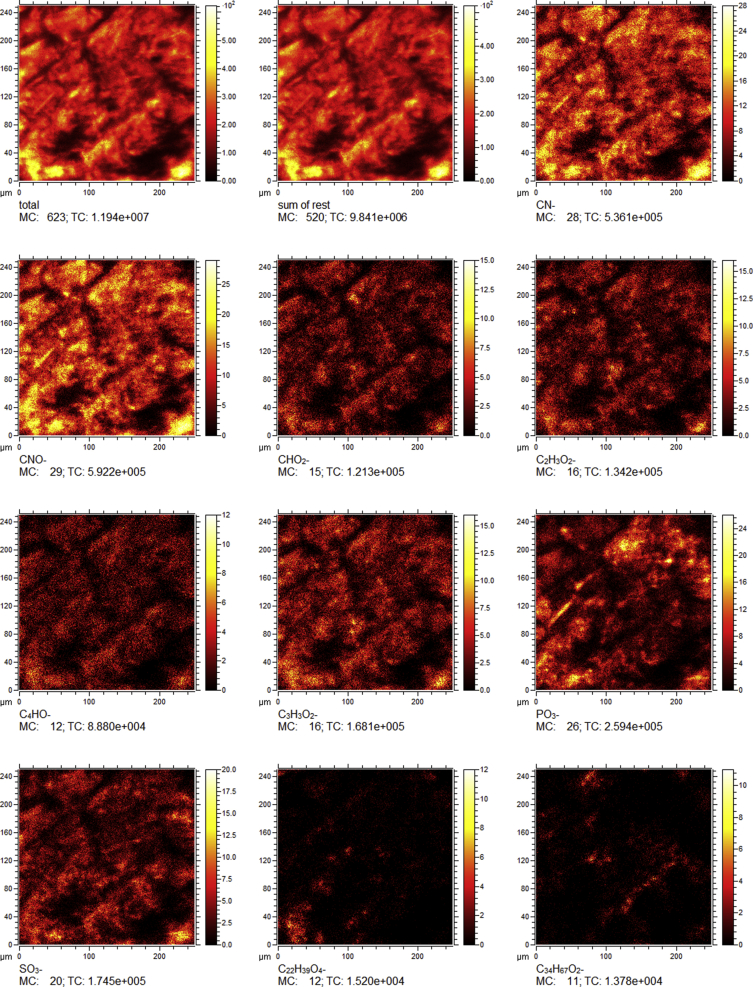


**Figure:**