# Supplementary figures and images for: Baseline characterization data for raw rice husk
Source: Data Brief. 2019 Jul 16;25:104219. doi: 10.1016/j.dib.2019.104219 (PMC6690668; doi:10.1016/j.dib.2019.104219)

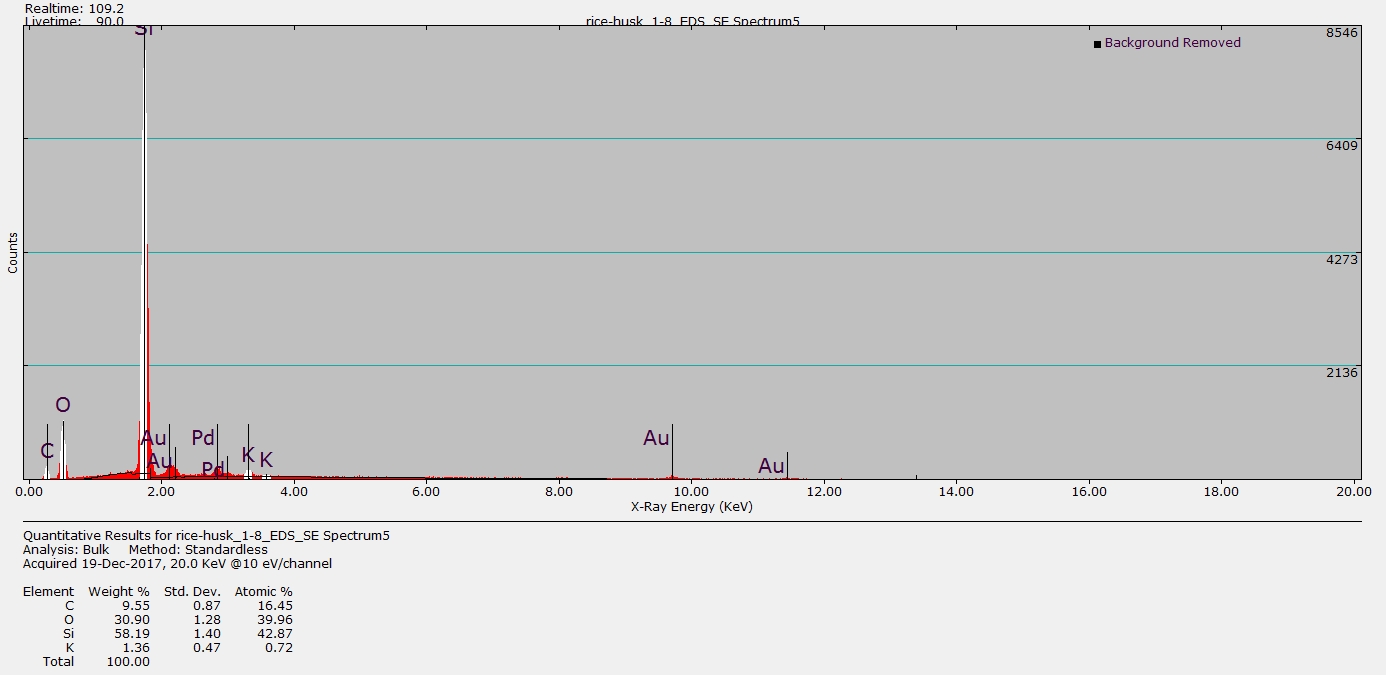

Supplement: Supplementary file 1 [file mmc1.zip › SEM Images/Rice Husk-EDX Spectra-Raw Data File-1.jpg]

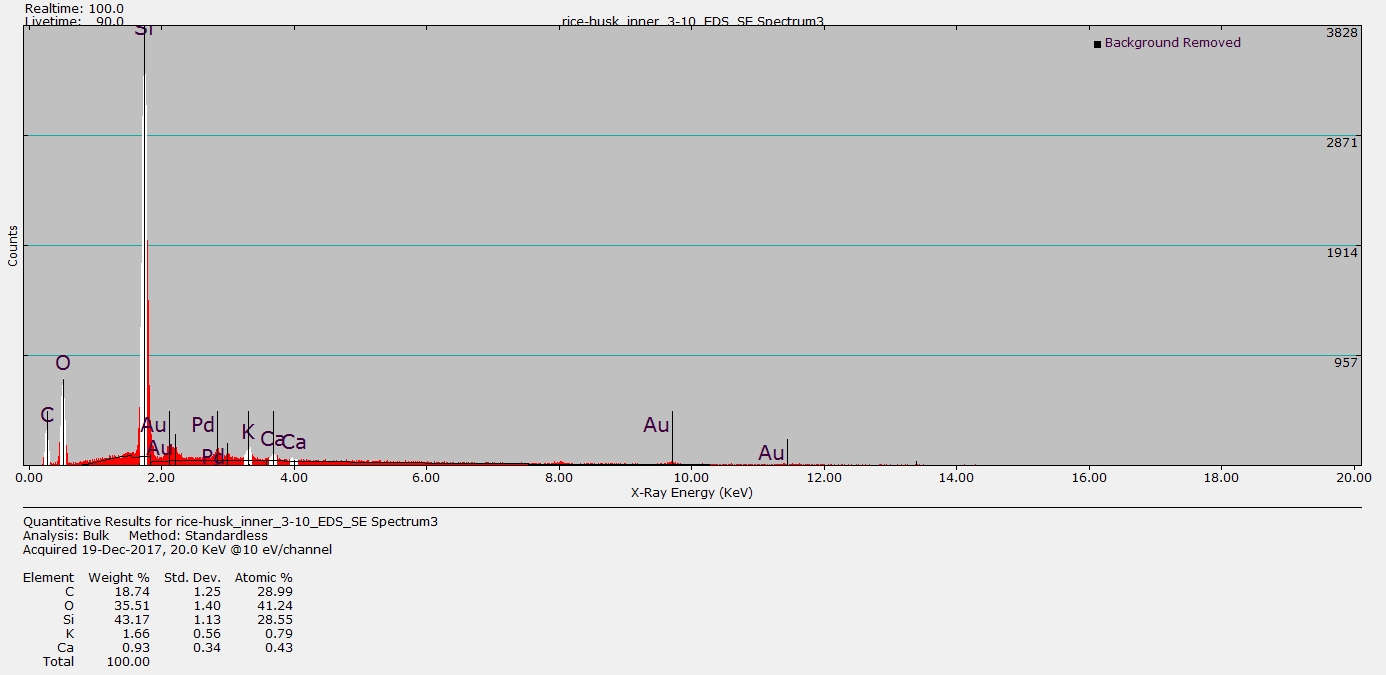

Supplement: Supplementary file 1 [file mmc1.zip › SEM Images/Rice Husk-EDX Spectra-Raw Data File-2.jpg]

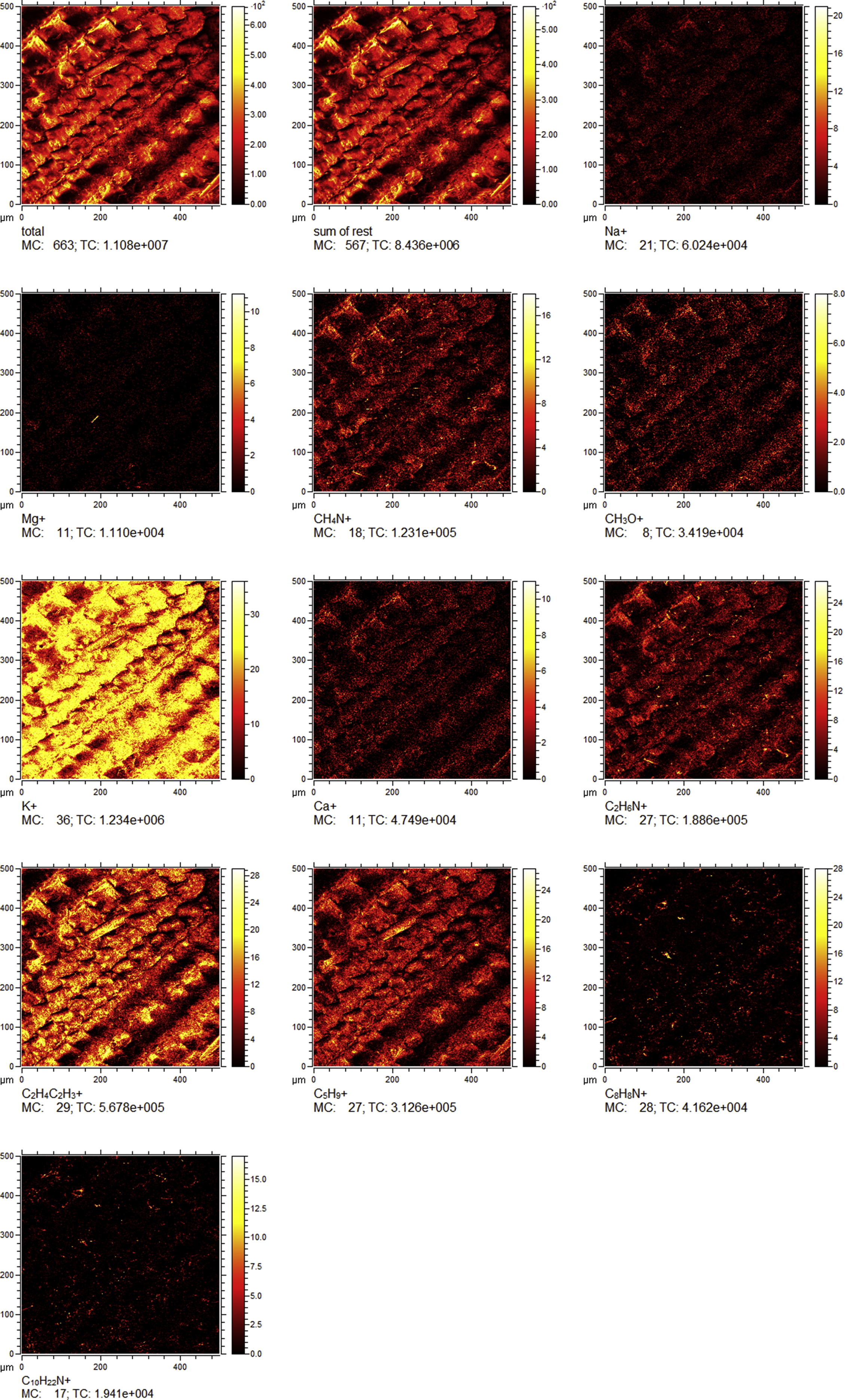

Supplement: Supplementary file 9 [file figs4.jpg]
