# Supplementary material for: Baseline characterization data for raw rice husk
Source: Data Brief. 2019 Jul 16;25:104219. doi: 10.1016/j.dib.2019.104219 (PMC6690668; doi:10.1016/j.dib.2019.104219)
Supplement: Supplementary file 4 [file mmc4.pptx]

## Slide 1
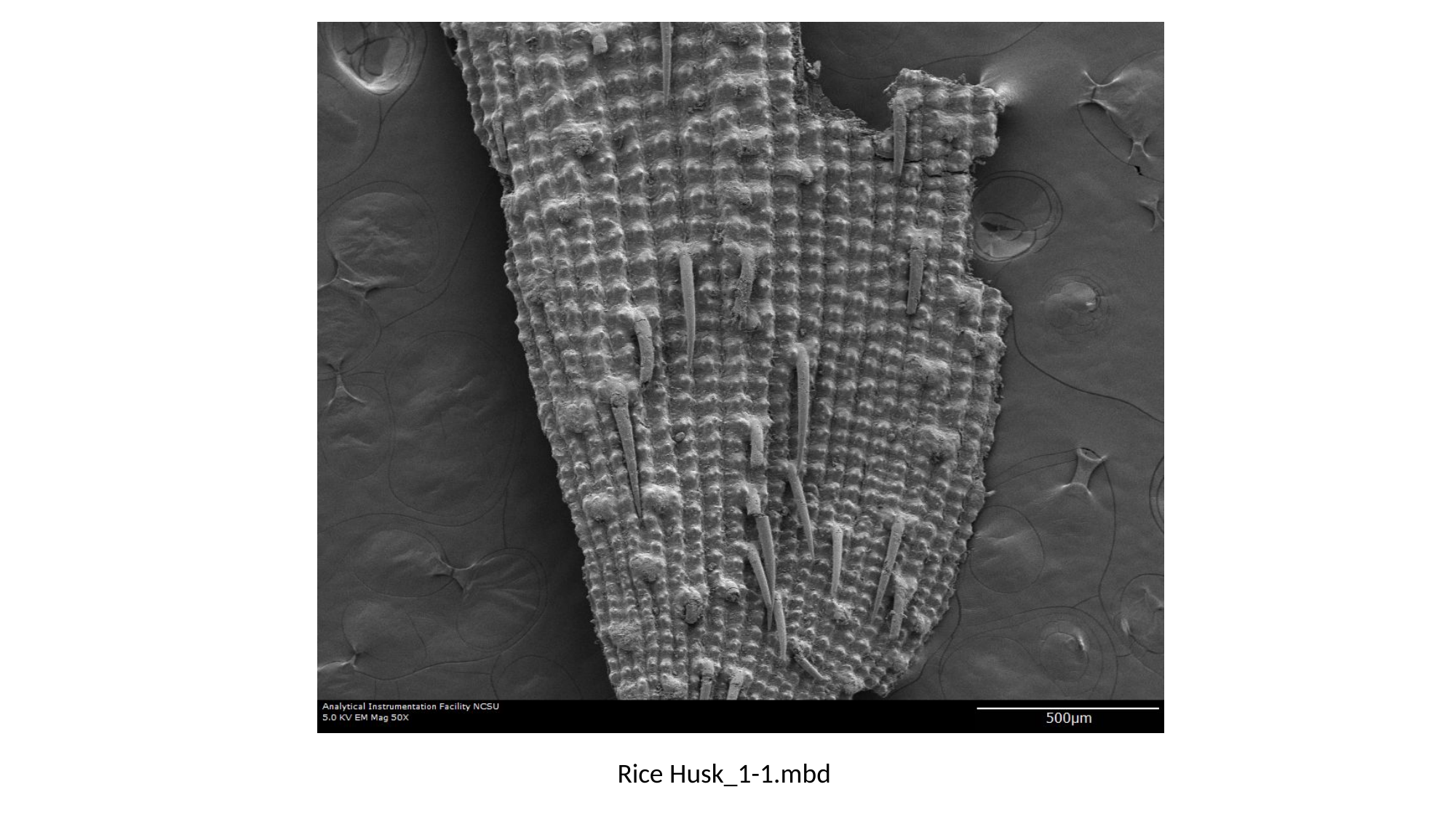

Rice Husk_1-1.mbd

## Slide 2
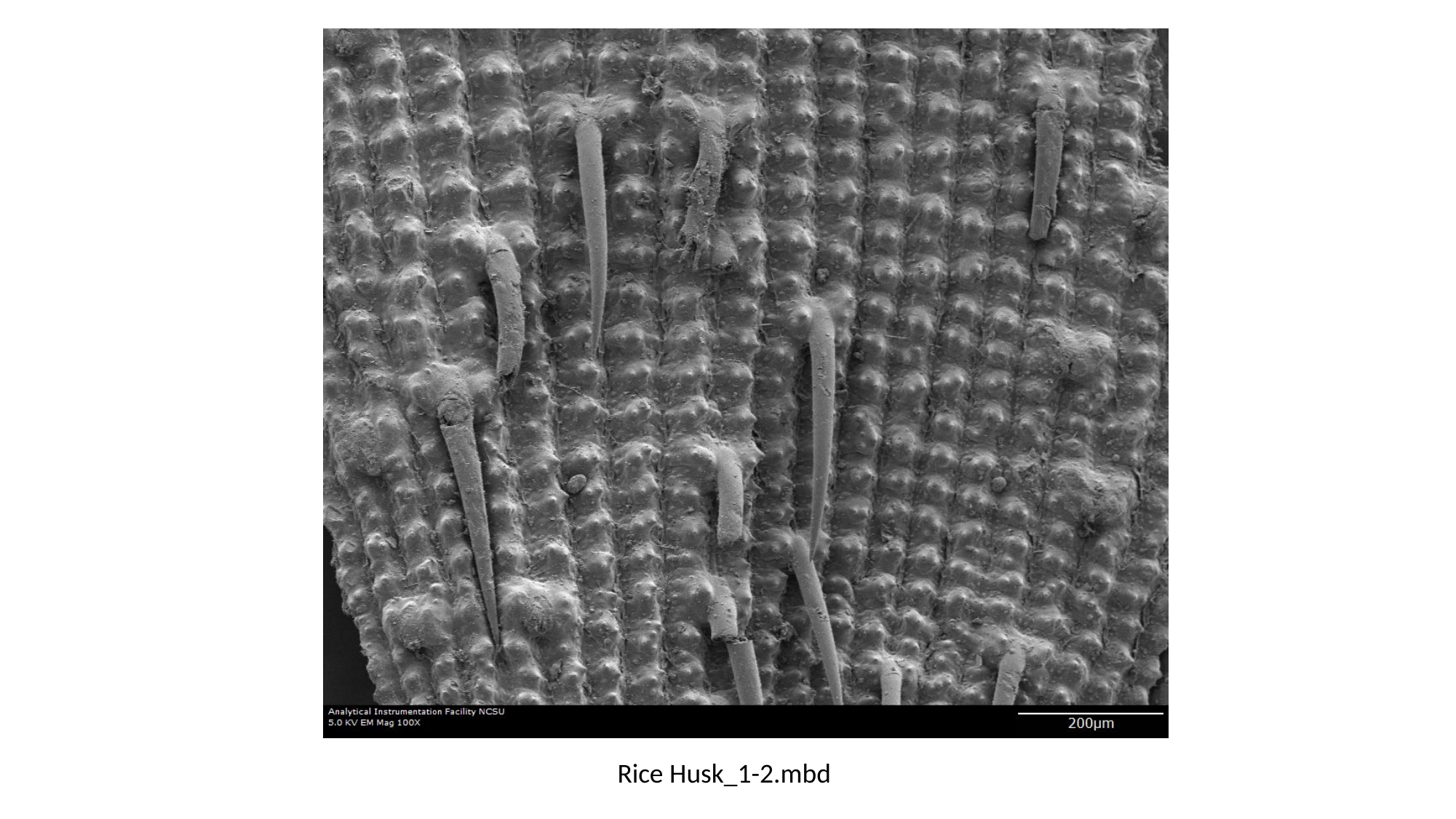

Rice Husk_1-2.mbd

## Slide 3
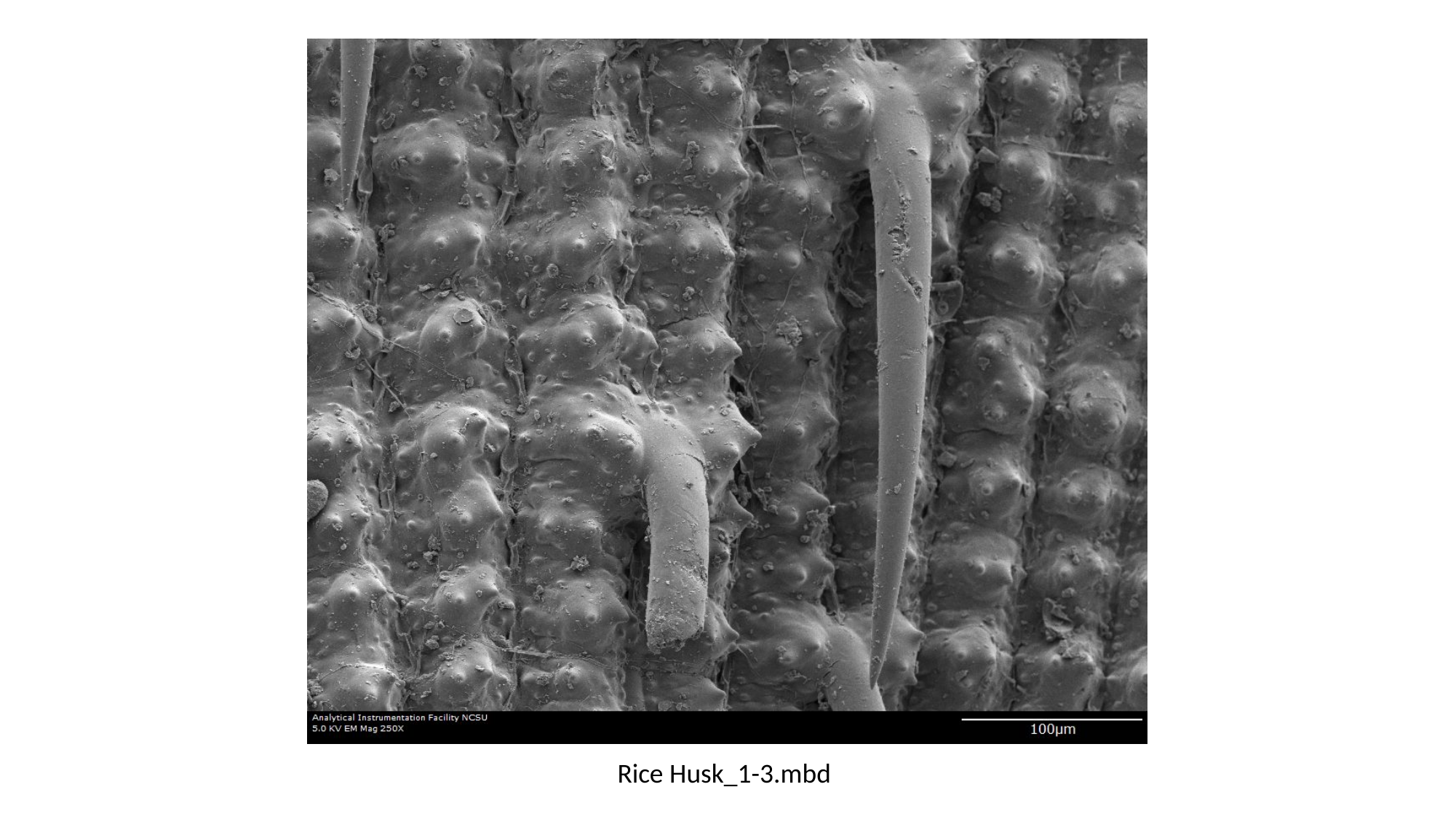

Rice Husk_1-3.mbd

## Slide 4
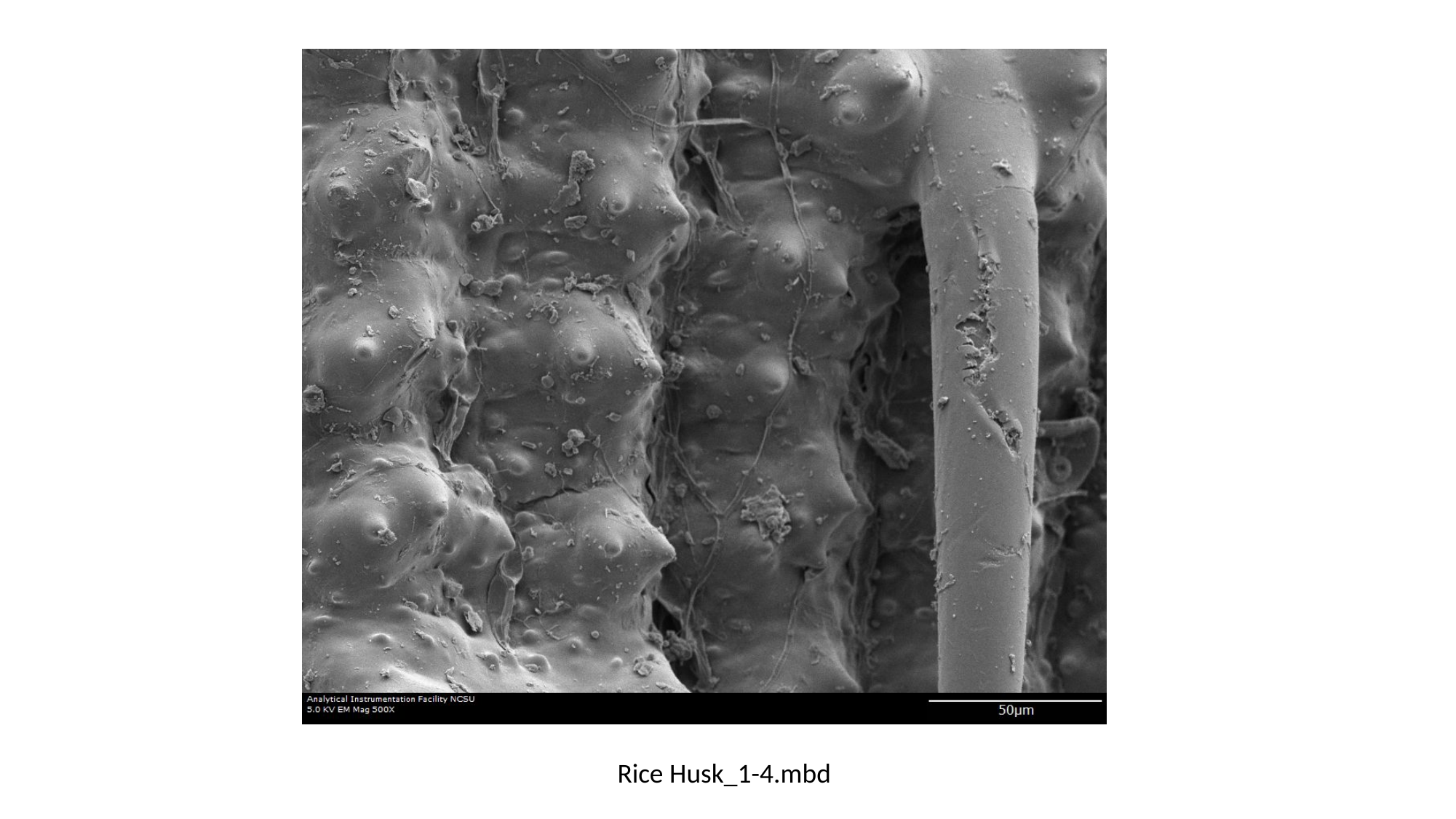

Rice Husk_1-4.mbd

## Slide 5
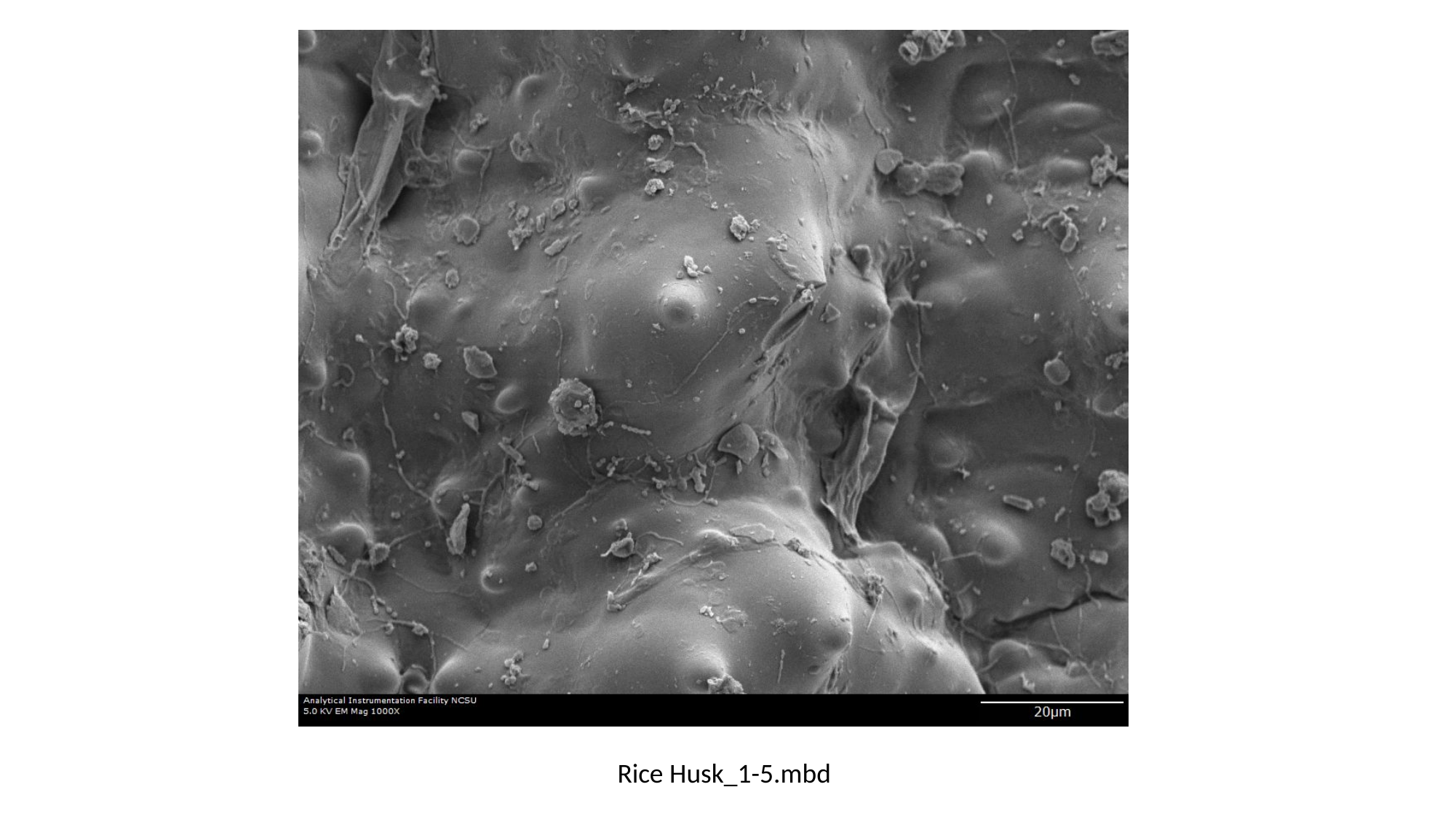

Rice Husk_1-5.mbd

## Slide 6
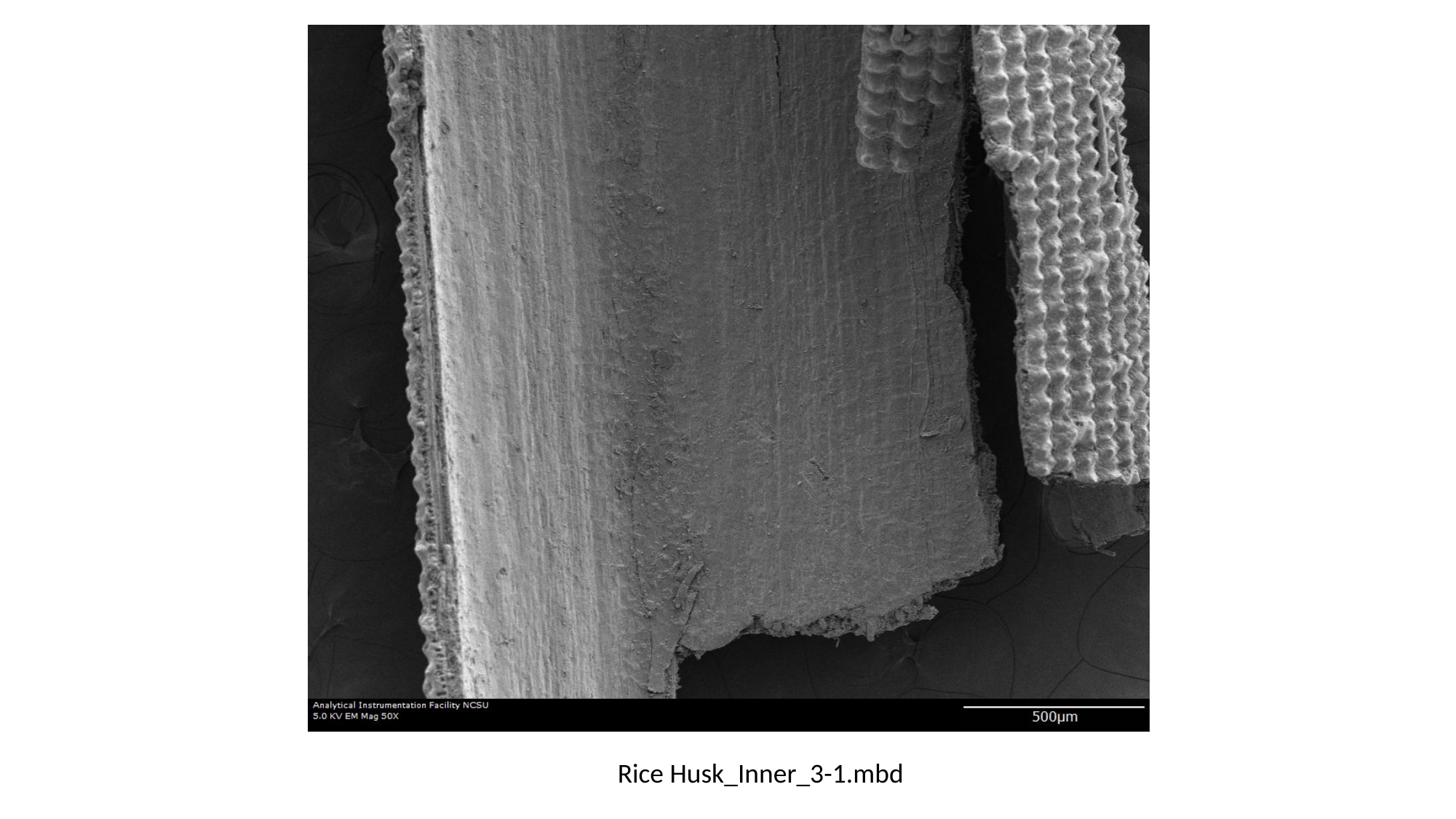

Rice Husk_Inner_3-1.mbd

## Slide 7
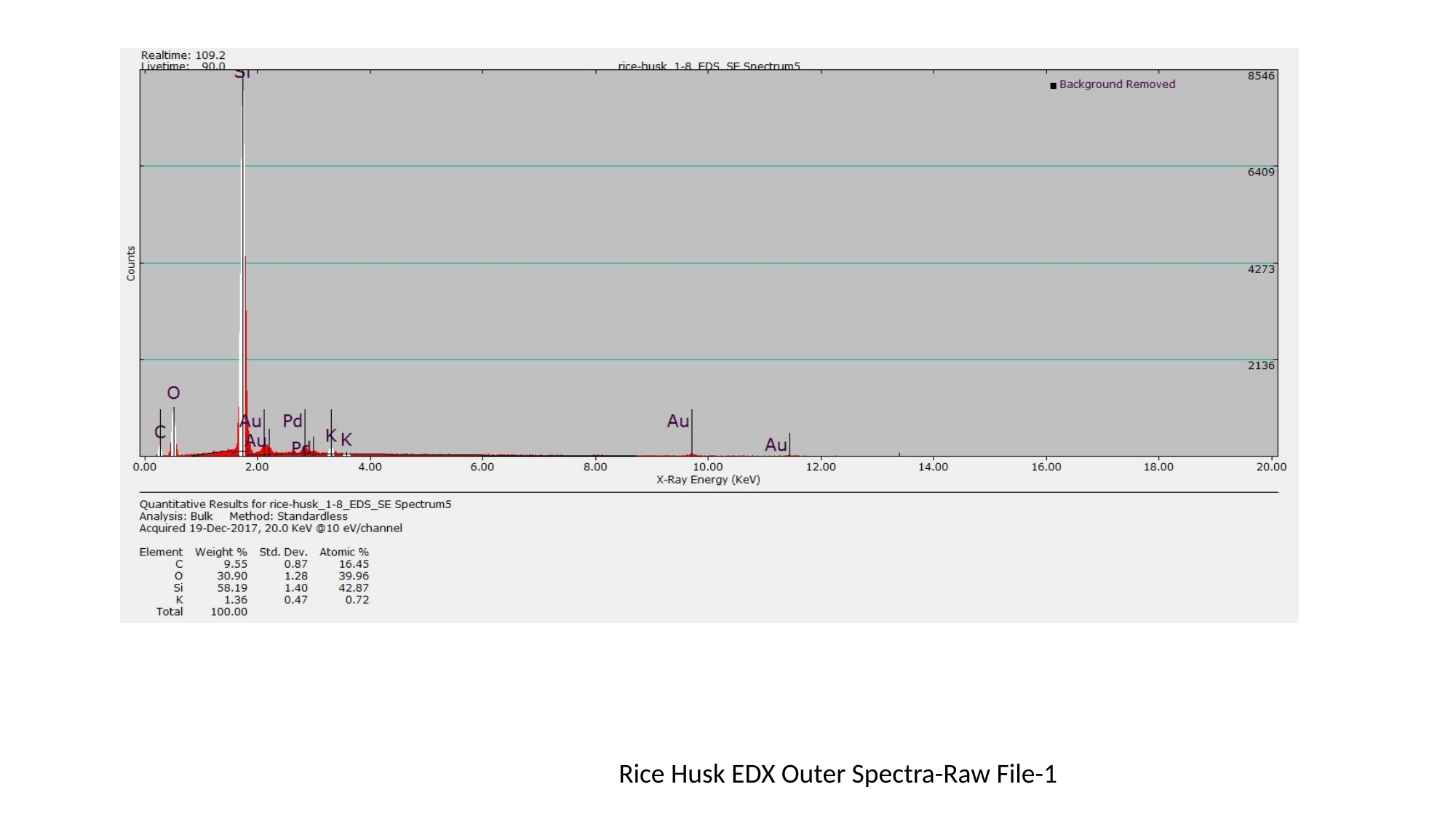

Rice Husk EDX Outer Spectra-Raw File-1

## Slide 8
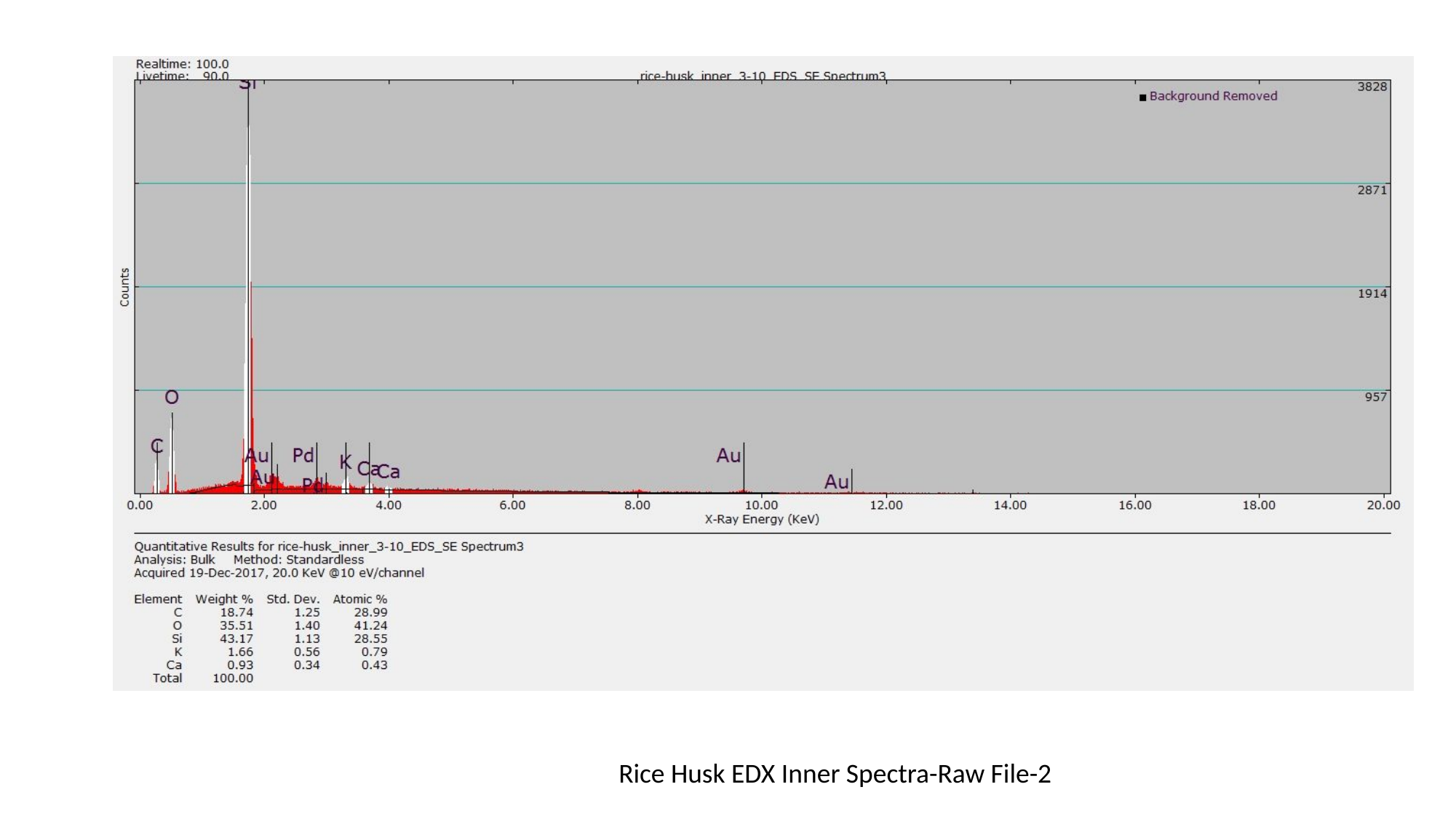

Rice Husk EDX Inner Spectra-Raw File-2
